# Editorial: Population and ancestry specific variation in disease susceptibility

**DOI:** 10.3389/fgene.2023.1267719

**Published:** 2023-09-20

**Authors:** Ranajit Das, Ekaterina A. Savina, Tatiana V. Tatarinova, Yuriy L. Orlov

**Affiliations:** ^1^ Yenepoya Research Centre, Yenepoya University, Mangalore, India; ^2^ The Digital Health Institute, I.M.Sechenov First Moscow State Medical University (Sechenov University), Moscow, Russia; ^3^ Engelhardt Institute of Molecular Biology RAS, Moscow, Russia; ^4^ Natural Science Division, La Verne University, La Verne, CA, United States; ^5^ Agrarian and Technological Institute, Peoples’ Friendship University of Russia (RUDN University), Moscow, Russia

**Keywords:** population genetics, ancestry, variation, disease sucseptibility, genomics

This Research Topic presents the studies in the field of human genetics and the genetic background of the diseases (https://www.frontiersin.org/research-topics/40281/population-and-ancestry-specific-variation-in-disease-susceptibility). It continues from “Association between Individuals’ Genomic Ancestry and Variation in Disease Susceptibility” Research Topic (https://www.frontiersin.org/research-topics/15891/association-between-individuals-genomic-ancestry-and-variation-in-disease-susceptibility).

As was stated in our previous Research Topic (Das et al., 2022), it is possible to glean precise ancestral origins using genetic information. Understanding one’s ancestry can play a monumental role in understanding variation in disease susceptibility across various populations and gleaning the complex gene and environment interplay in ancestry-specific disorders that may include cancers. Recently, population-specific variations in cancer types have been shown. For instance, cardiovascular diseases tend to manifest in distinct ways unique to the ancestry of the patient as was discussed for cardiovascular disease in African ancestry populations (Harshfield et al., 2021). Traditional high fat and protein diets in cold regions of North Asia have consequences on obesity and diabetes-related diseases (Bai et al., 2015; Tiis et al., 2020). Ancestry associations with obesity in the Arab population in Kuwait were discussed by Dashti et al. (2021). Zinchenko et al. (2021) and Petrova et al. (2021) discussed point and cumulative prevalence, as well as the burden of rare hereditary diseases in Russia based on sequencing technologies. In addition, COVID-19 has also been shown to have population and ancestry-specific variations (Gozman et al., 2021). These works are presented in our edited Research Topic on ancestry in disease susceptibility (https://www.frontiersin.org/research-topics/15891/association-between-individuals-genomic-ancestry-and-variation-in-disease-susceptibility).

The current Topic complements the recent Research Topic series “Bioinformatics of Genome Regulation” (https://www.frontiersin.org/research-topics/40408/bioinformatics-of-genome-regulation-and-systems-biology-volume-iii) in *Frontiers in Genetics* that considered genetic backgrounds rather than molecular mechanisms of the human diseases (Orlov et al., 2021a; Anashkina et al., 2023). The articles published in this Research Topic extend the studies presented in *Frontiers in Genetics* Topics on gene expression regulation and high-throughput sequencing in chronic disease markers (https://www.frontiersin.org/research-topics/53085/high-throughput-sequencing-based-investigation-of-chronic-disease-markers-and-mechanisms-volume-ii) (Orlov et al., 2022). Recently, we had a series of publications on human ancestry based on genomics data (Das et al., 2020; Orlov et al., 2021b) that were associated with the BGRS\SB (Bioinformatics of Genome Regulation and Structure\Systems Biology) conference series and bioinformatics studies (Orlov et al., 2016; Orlov and Baranova, 2020; Anashkina et al., 2021) that have been widely presented at *Frontiers in Genetics* Research Topics since 2014 (Orlov et al., 2015).

Overall, this Research Topic collected four articles (Al Madhoun et al., 2022; Cullina et al., 2023; James et al., 2023; Sezgin and Kaplan, 2022).

James et al. review genetic ancestry as a risk factor for the incidence of non-small cell lung cancer in the US. There are known racial and ethnic differences in lung cancer risk, survival, and mortality in African Americans, Caucasian Americans, Hispanic Americans, and Latin Americans (Siegel et al., 2022). Despite lower smoking prevalence, African Americans experience the highest burden of lung cancer in comparison to Caucasian Americans. This literature review suggests that differences in non-small cell lung cancer risk, incidence, and survival in ethnic populations may be more likely attributable to general lifestyle, behavioral, and environmental factors.

Madhoun et al. discuss a genetic variant of metabolic syndrome in Arab and Asian ethnic cohorts. Metabolic syndrome is triggered by various factors that include genetic predisposition, aging, obesity, insulin resistance, and physical inactivity (Tiis et al., 2020). One of the genetic risk loci is the Caveolin-1 gene. The global epidemic proportions of metabolic syndrome are estimated from 12% to 37% in the Asian populations. The authors find ethnic variation in Caveolin-1 in the Kuwaiti Arab cohort.

Sezgin and Kaplan analyze Behçet disease (BD) susceptibility. It is a multisystem arterial and venous inflammatory condition with wide clinical spectrum manifestations. Behçet disease is mostly seen in populations from East Asia to the Mediterranean, therefore, it is referred to as the so-called “Silk Road disease.” The authors identify a small number of BD risk genes with unique evolutionary histories in East Asians.

Cullina et al. study the risk loci for peripheral artery disease in a Dominican population. This form of atherosclerotic cardiovascular disease is known to have racial and ethnic disparities. The authors identify a Native American ancestry tract at chromosome 2q35 that is significantly associated with peripheral artery disease.

Thus, this Research Topic presents a series of research works on population-specific variation in human diseases (Al Madhoun et al., 2022; Cullina et al., 2023; James et al., 2023; Sezgin and Kaplan, 2022). The complementary Research Topic in *Frontiers in Genetics* named “High-throughput Sequencing-based Investigation of Chronic Disease Markers and Mechanisms, Volume II” (https://www.frontiersin.org/research-topics/53085/high-throughput-sequencing-based-investigation-of-chronic-disease-markers-and-mechanisms-volume-ii) continues the collection of articles on the markers of human diseases (Orlov et al., 2022).
